# Supplementary material for: Repeat-based holocentromeres of the woodrush Luzula sylvatica reveal insights into the evolutionary transition to holocentricity
Source: Nat Commun. 2024 Nov 5;15:9565. doi: 10.1038/s41467-024-53944-5 (PMC11538461; doi:10.1038/s41467-024-53944-5)
Supplement: Supplementary file 1 — Supplementary Information [file 41467_2024_53944_MOESM1_ESM.pdf]

Repeat-based holocentromeres of the woodrush *Luzula sylvatica* reveal insights into the evolutionary transition to holocentricity

Mata-Sucre *et al.*

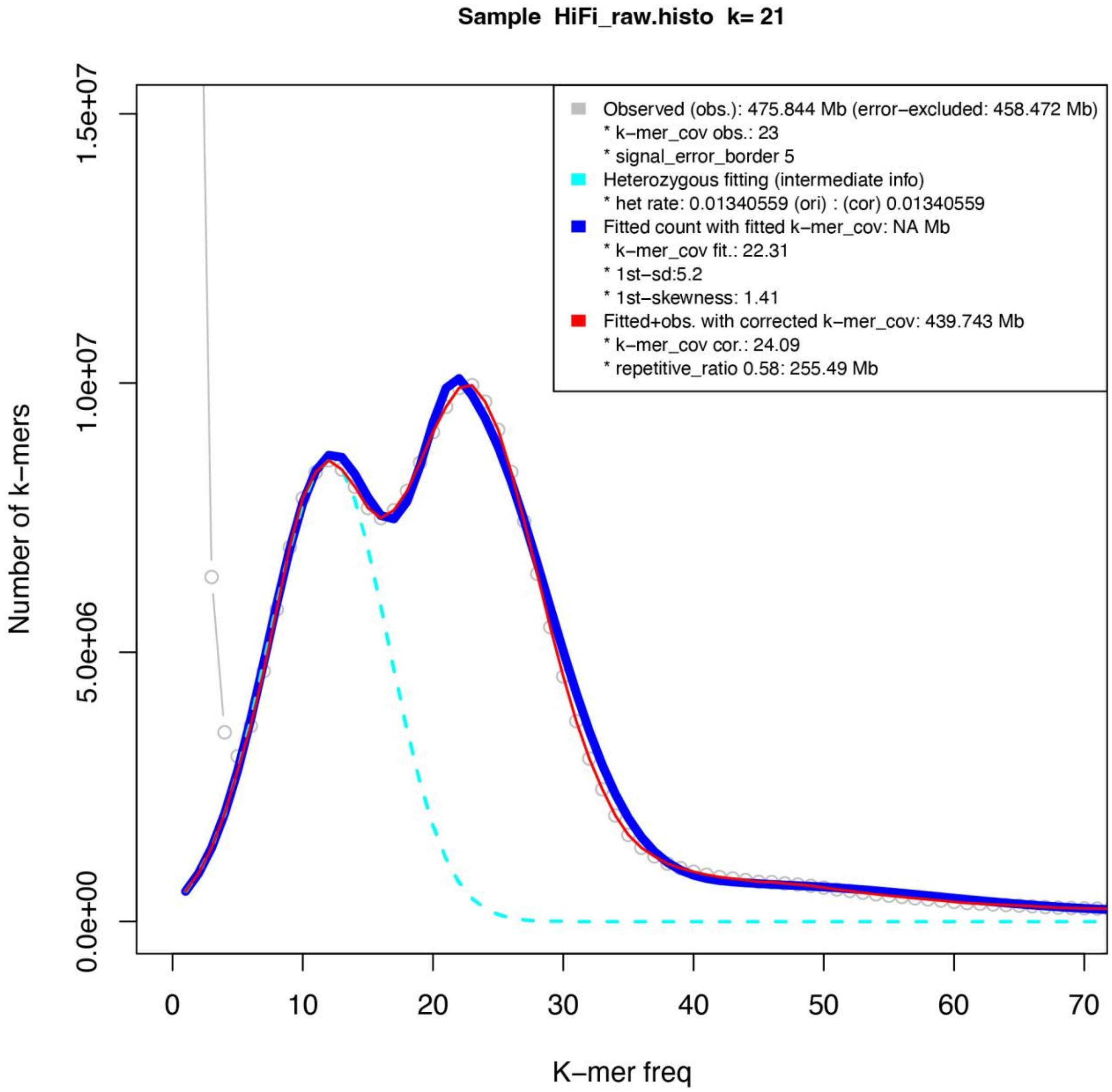

Supplementary Figure 1. findGSE K-mer-based genome size estimation of *Luzula sylvatica*.

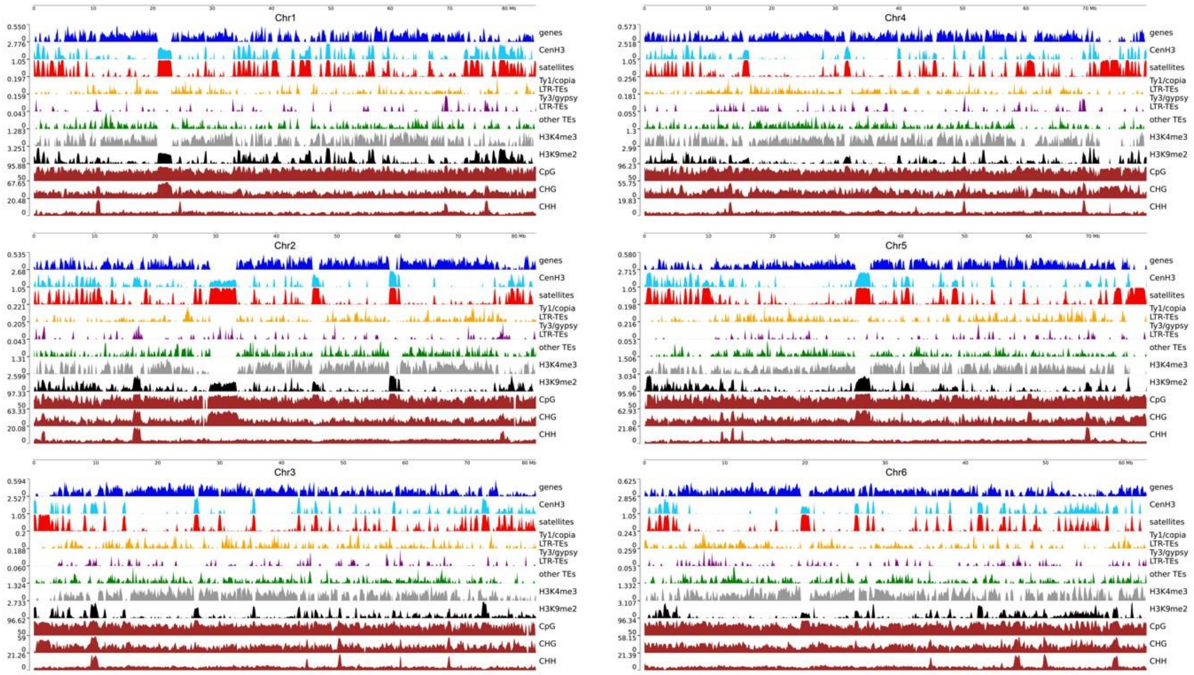

Supplementary Figure 2. Detailed view of *Luzula sylvatica* chromosomes showing the dispersed distribution of the main genomic features. CENH3, gene, tandem repeat, dispersed repeat, eu-heterochromatin and histone mark densities, typical of holocentric chromosomes. ChIPseq signals are shown as log2 (normalized RPKM ChIP/input). Methylation signals are shown as a percentage of methylated bases in each (CpG, CHG, CHH) context. Gene, satellite, and TE densities shown as proportion in each window. Window sizes of 100 kb.

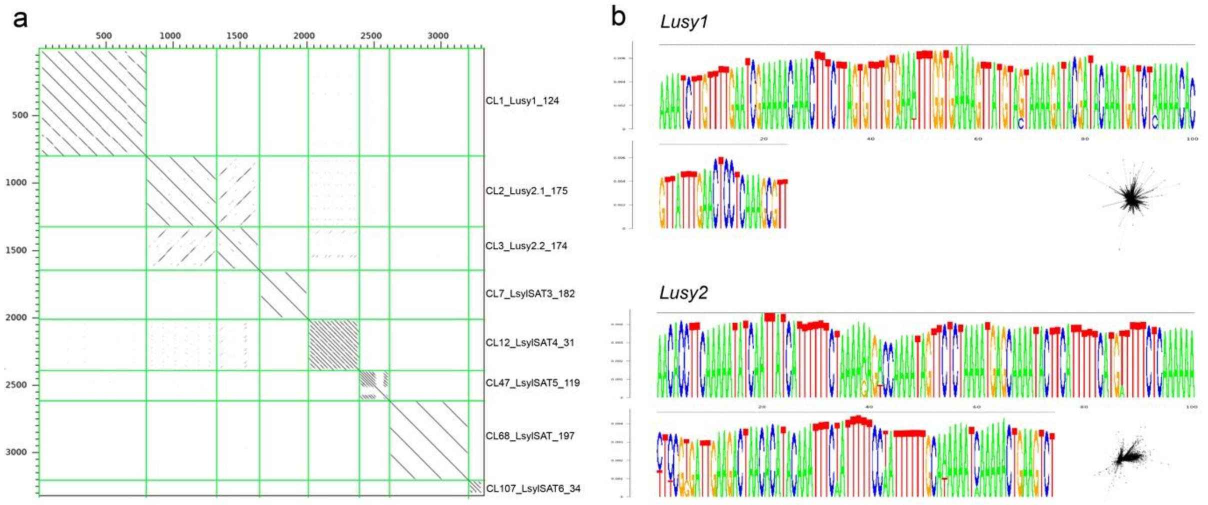

c

```
Consensus  TTGTGTGWRYWMSTYYWMAAWTAYSRAKNANNATNTTDTTTTGAATAGAACAAVAAAVTTBHBTADTDTGHGTGAAAT--ACTTCANTTTTNANNTTTT 97
Lusy1      ----------------------------AAAATCTTGTTTTGAATCGAAAAACAAACTTTCTTAGTGTTTGTGAAA-------------------TT 50
Lusy2.1    TTGTGTGTGTTAGTCTTCAATTACGGAG--------TATTTTGAGTACGACATGAAAATTGAGTATTTTGCTTGTGTTACACTTCAATTTTAACATTTT 91
Lusy2.2    -------AACACCTTCAAAAATATCAATTATCATTTTTTCTAAAAAAGACCAAAATAGTTCTCTAAAATGAGCAAAAT--ACTTCATTTTTCATGTTTT 90

Consensus  TBTHAAAAADHTCTGTADTDAAAGAT-TDAHACATATDANNNTNNANTTTTNNNATTTTTTVBAAAAAHTC--AAAAATTGAAVTCYTCAAAGCGTT 191
Lusy1      TGGTGAAAAGTTATGTAGTAAAAGATACGATACAAATGA---------------------TCTAAAAACAC--GTTATTTGAACTCCTCAAAGCGTT 124
Lusy2.1    TTTAAAAAATATCTTAATTGAATTTT-TTGAAGGTGTTAGACTTCAGTTTTTGCATTTTTAAGAAATTTTTGAAAAAAATTGAAAT------------ 175
Lusy2.2    TCTCAAAAAACTCCGTGATTGAAGAC-TAACACATACAAATTTCTATTTTTCCTATTTTTTGCAAAAAATC--AAAAACTGAAGTCT---------- 174
```

Supplementary Figure 3. *Luzula sylvatica* satellite characterization. (a) Dot plot showing similarities between groups of tandem repeats. Despite being classified as a tandem pattern, LsylSAT_197 was not considered as a satellite because the genome mapping did not show a tandem distribution. **(b)** Sequence logo the most abundant satellite clusters LsylSatCL1 (*Lusy*1) and LsylSatCL2 (*Lusy*2). **(c)** Alignment between the two variants of *Lusy*2 (LsylvSat174 and LsylSat175) and *Lusy*1.

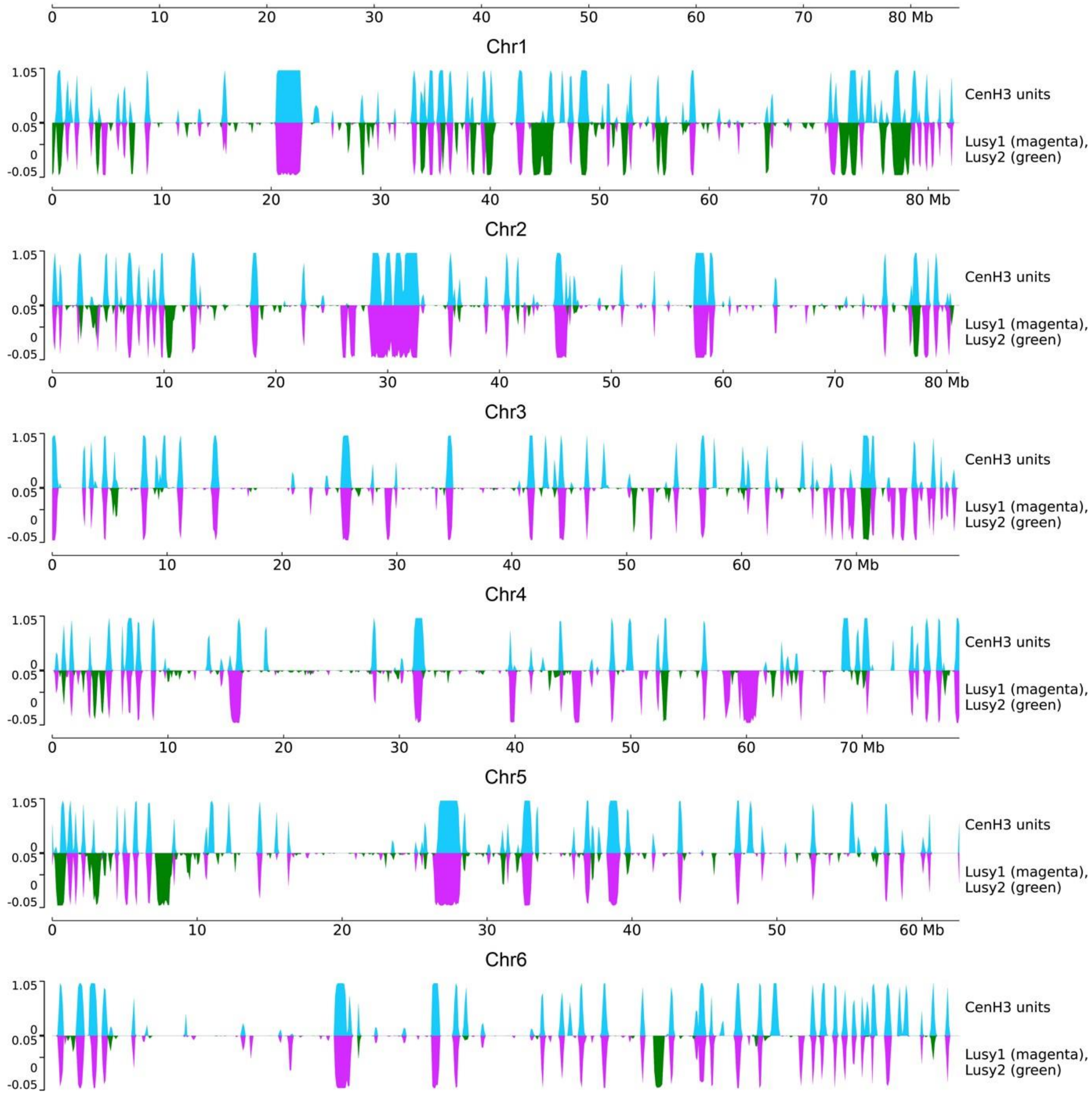

Supplementary Figure 4. Proportion of CENH3 units (light blue), *Lusy1* (magenta) and *Lusy2* (green) arrays in 100kb windows.

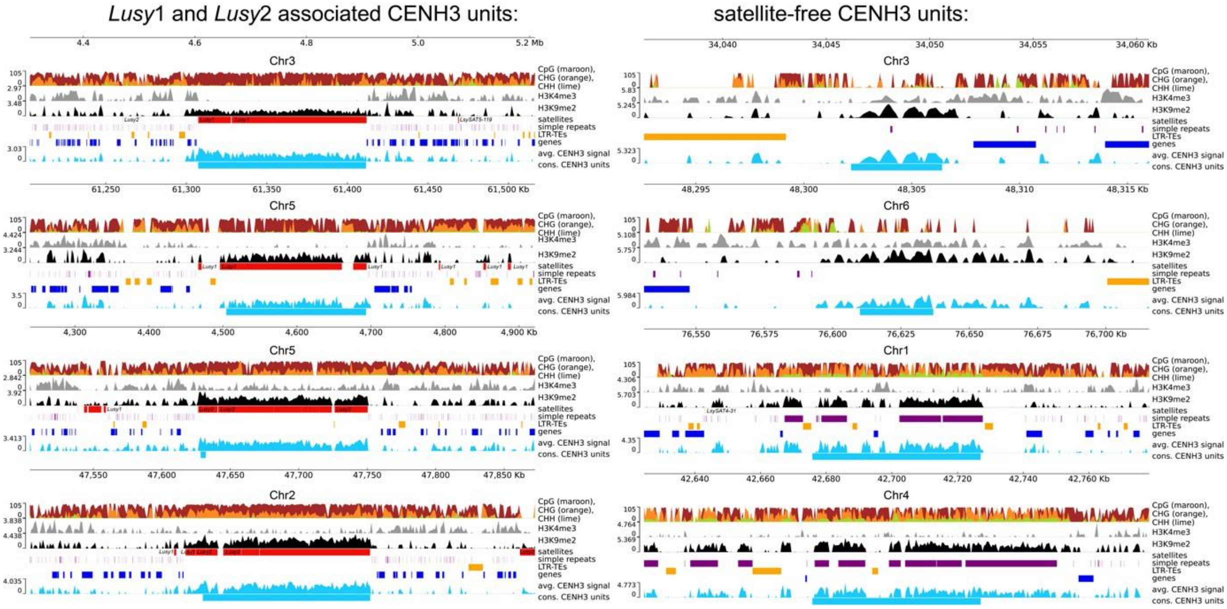

Supplementary Figure 5. Examples of (epi)genetic features distribution near satellite-based and satellite-free CENH3 domains of *L. sylvatica*. ChIPseq signals are shown as log2 (normalized RPKM ChIP/input). Methylation signals are shown as a percentage of methylated bases in each (CpG, CHG, CHH) context.

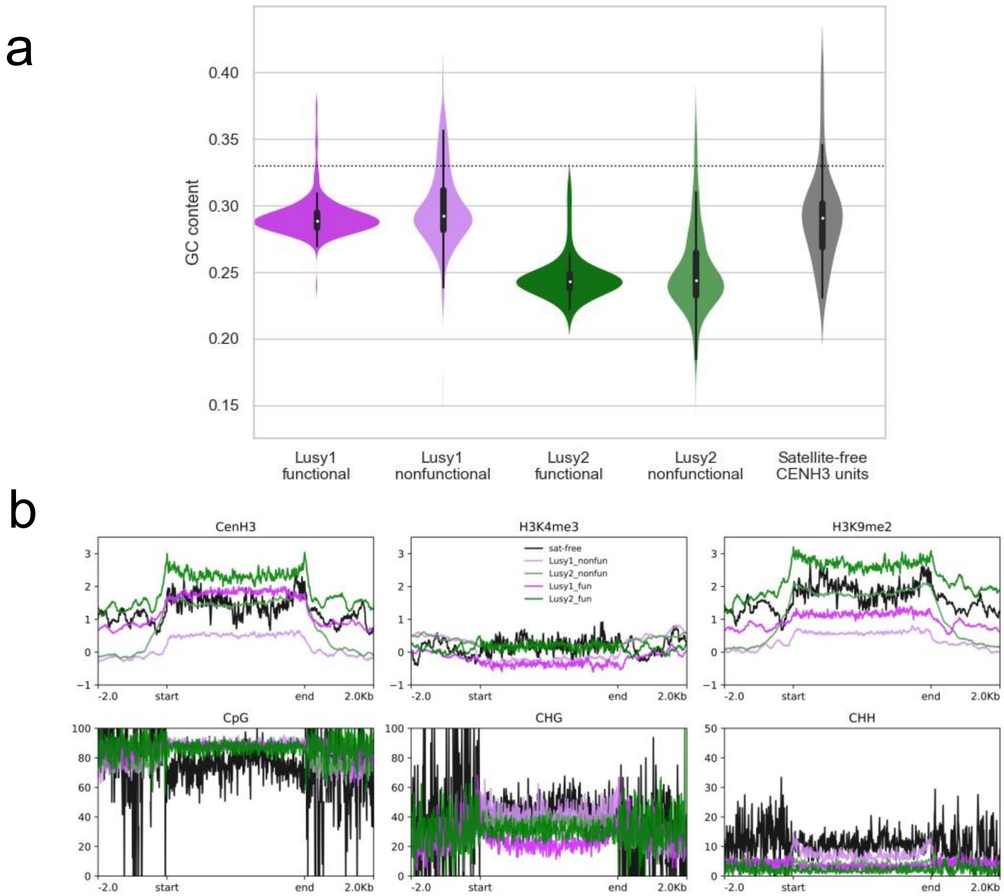

Supplementary Figure 6. Comparative analysis of GC content and epigenetic markers in functional/nonfunctional *Lusy* satellite arrays and satellite-free CENH3 domains. (**a**) GC proportion of functional and nonfunctional satellite arrays and satellite-free CENH3 units. Dotted line at the overall genomic GC content level. Inner boxes of violin plots follow the definition in seaborn data visualization package where central points represent median value, boxes represent 1st and 3rd quartiles, and whiskers represent the data range without outliers, defined as observations further than 1.5 of interquartile range from the respective (1st or 3rd) quartile. The number of observations (array count) is 247, 457, 107, and 845 for functional Lusy1, nonfunctional Lusy1, functional Lusy2, and nonfunctional Lusy2, respectively. (**b**) Metaplots showing the level of enrichment of functional and nonfunctional satellite arrays and *Lusy* satellite-free CENH3 units with CENH3, H3K4me3, H3K9me2, CpG, CHH, and CGH. ChIPseq signals are shown as log2 (normalized RPKM ChIP/input). Methylation signals are shown as a percentage of methylated bases in each (CpG, CHG, CHH) context. Source data are provided as a Source Data file.

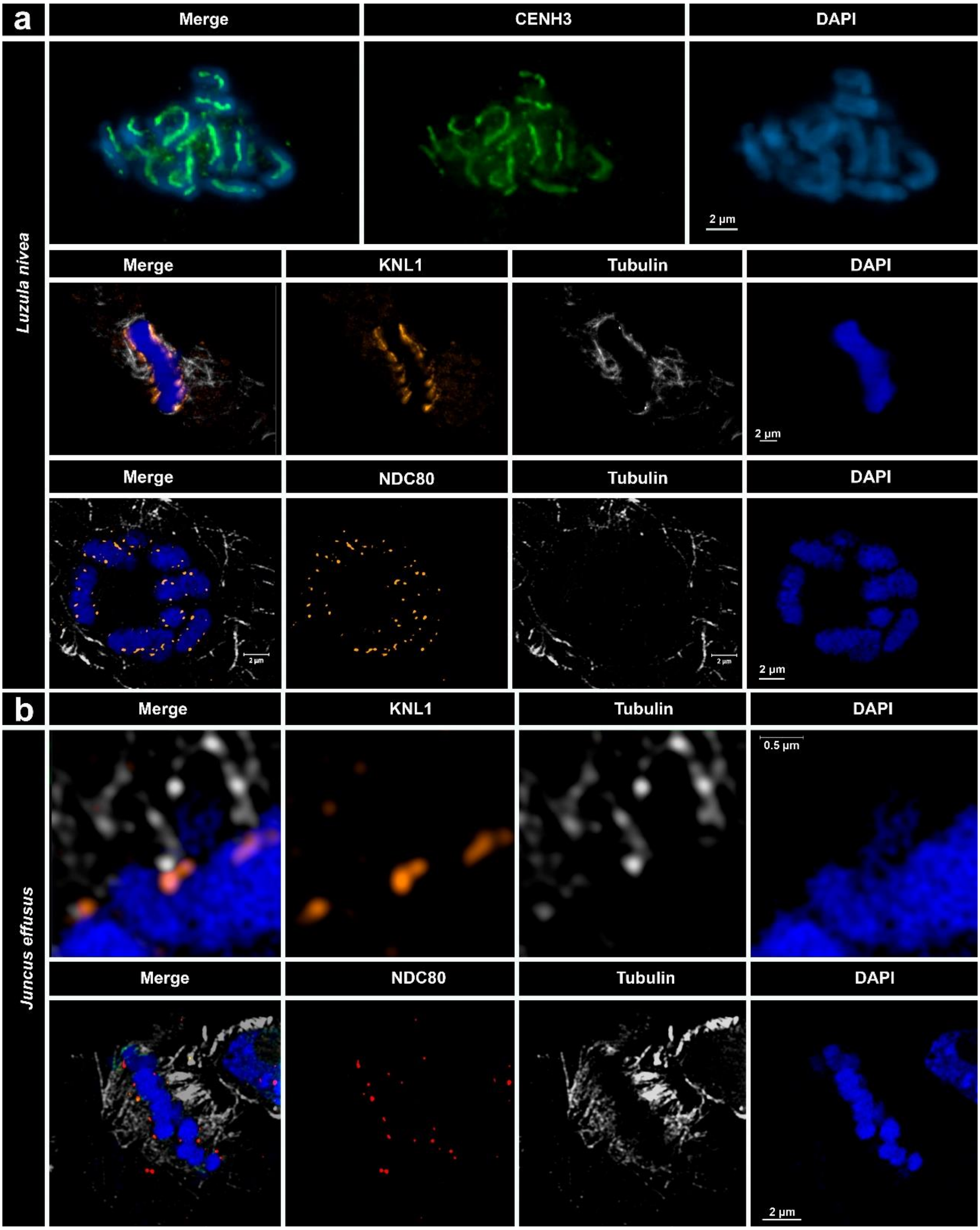

Supplementary Figure 7. Localization of CENH3, KNL1, NDC80 and tubulin in *Luzula nivea* (a) and *Juncus effusus* (b) metaphase chromosomes. KNL1 and NDC80 localize specifically to the surface of the *J. effusus* centromere, where microtubules attach (n = 10). The images in **(b)** represent super-resolution (a single slice of a 3D-SIM image stack).

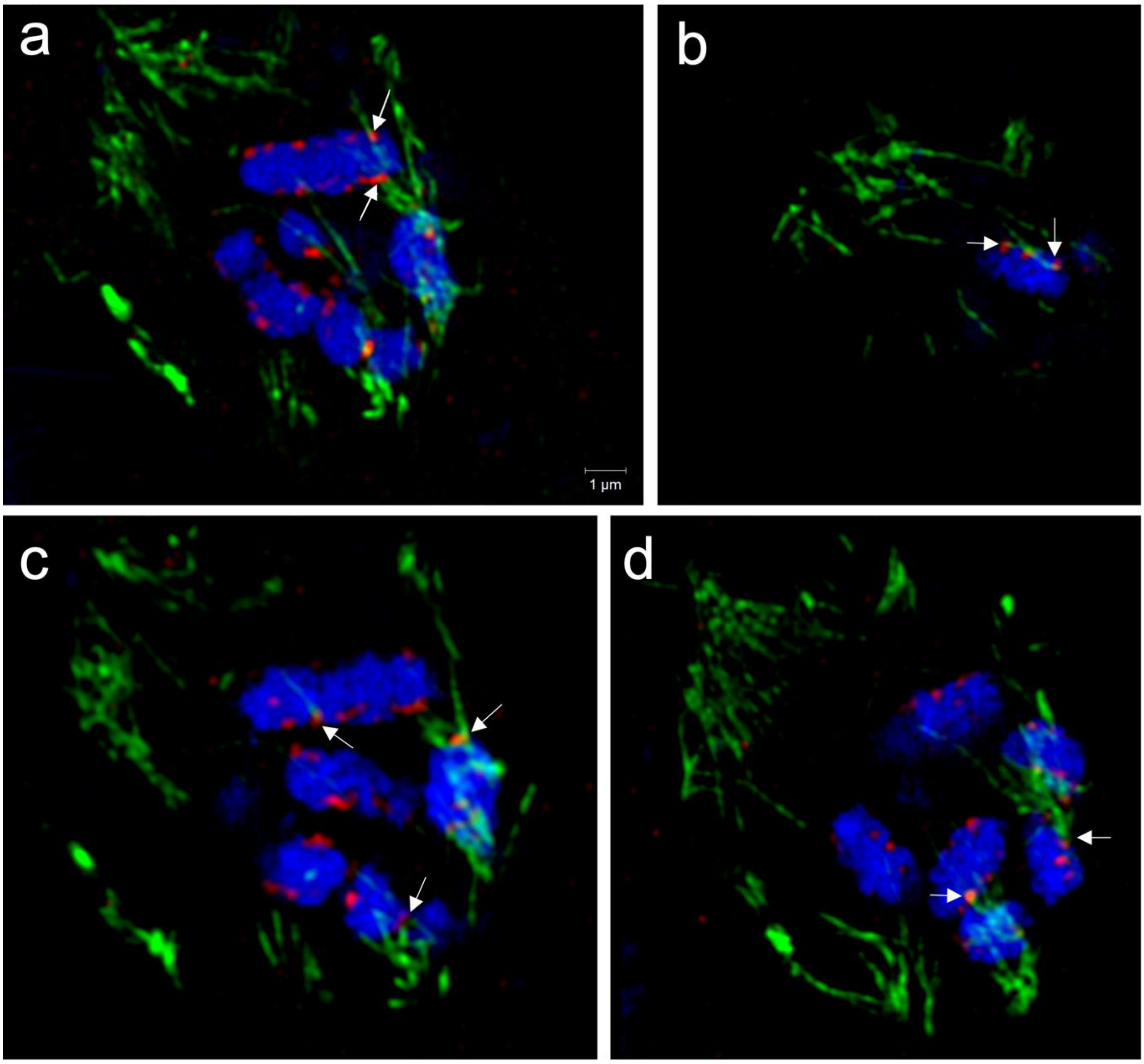

Supplementary Figure 8. Localization of KNL1 and tubulin in *Luzula sylvatica* mitotic chromosomes. The images in **(a–d)** represent 3D-SIM super-resolution differential plane sections of the Suppl. Movie 1. Please note the interaction between KNL1 and tubulin (indicated by arrows; n = 5).

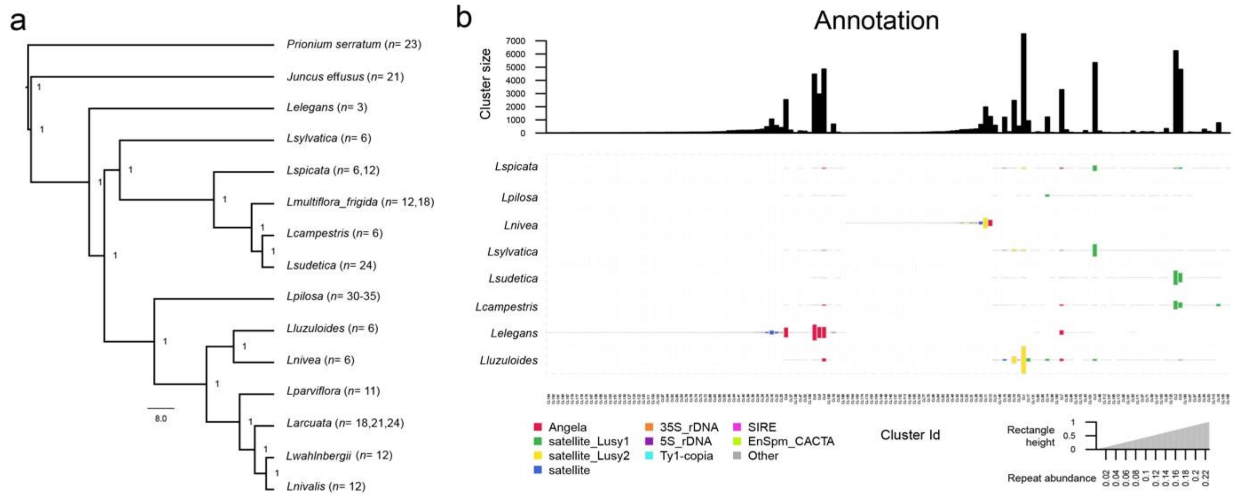

Supplementary Figure 9. Phylogenetic relationships and comparative repeat analysis among *Luzula* species. (**a**) Whole-plastome phylogenetic analysis. The plastome of *Juncus effusus* (MW366789) and *Prionium serratum* (OL689155) were used as outgroup. Chromosome numbers were recorded here and compiled from Záveská Drábková (2013). (**b**) Comparative abundance of the main types of repetitive sequences in *Luzula* species. The size of the rectangle is proportional to the genome abundance of that cluster for each species. The colors of the balls correspond to different repetitive sequence types. The proportion of each cluster was adjusted according to genome size. Source data are provided as a Source Data file.

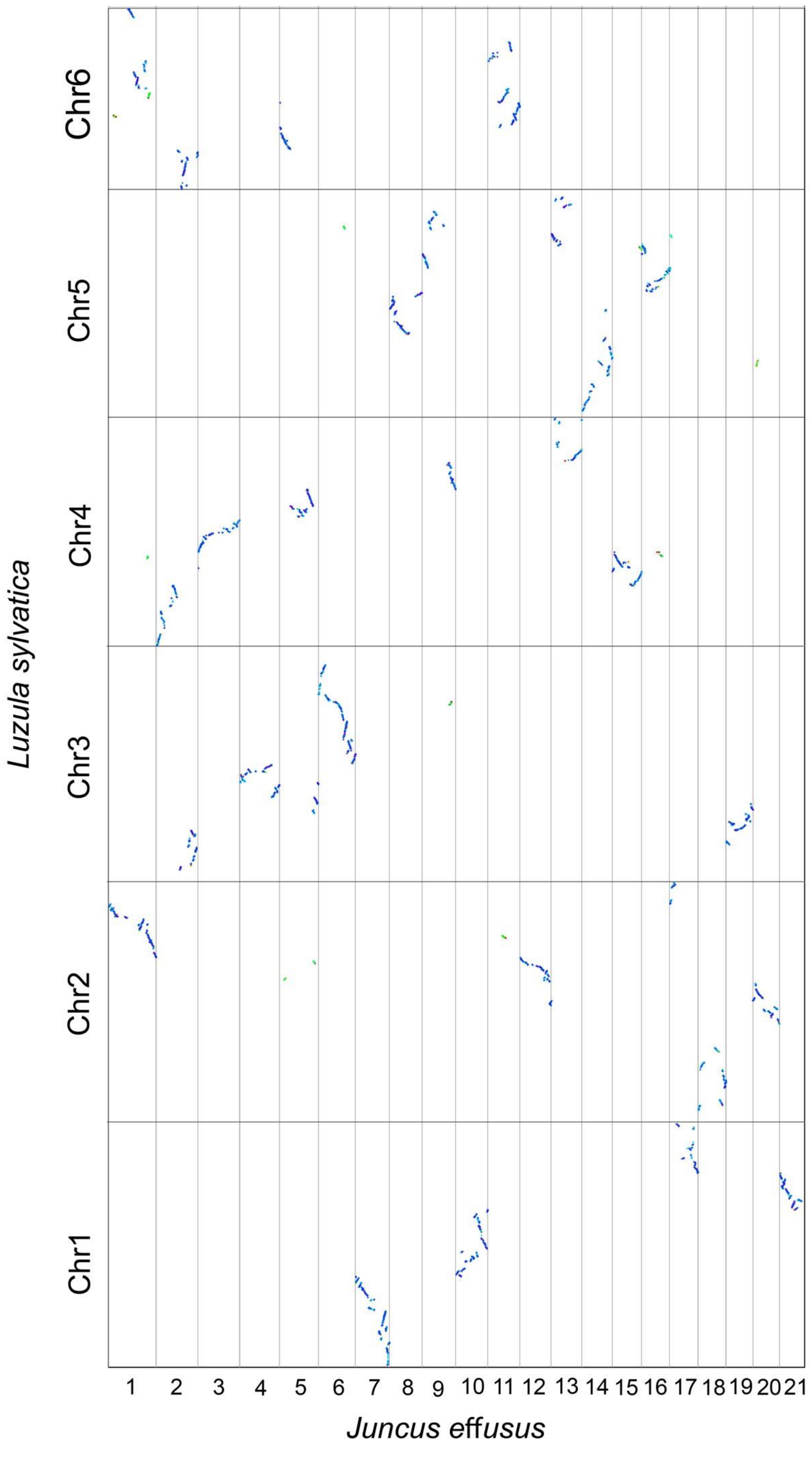

Supplementary Figure 10. Genome synteny patterns showing macro-conserved blocks of *J. effusus* that are part of the *L. sylvatica* chromosomes.

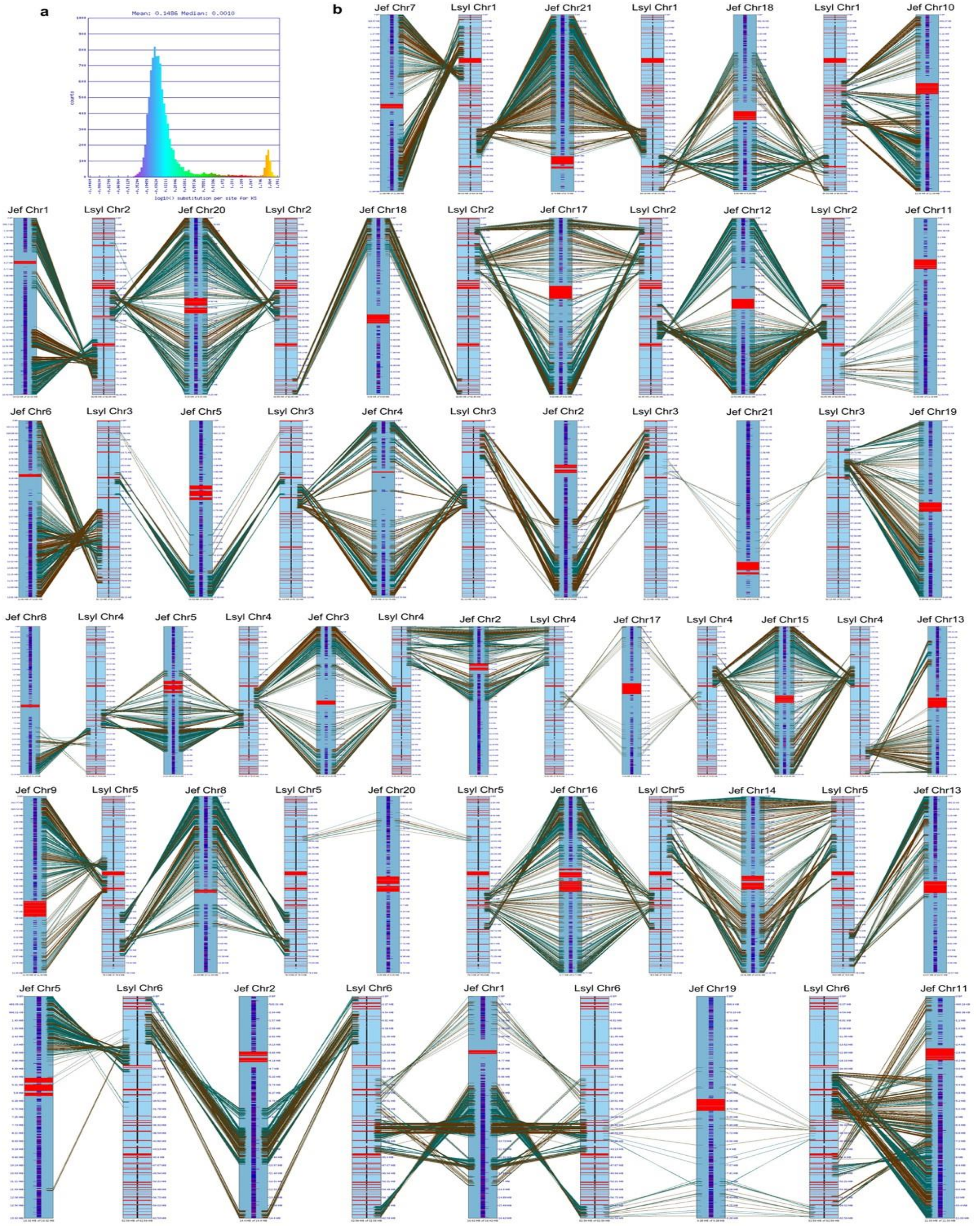

Supplementary Figure 11. ***L. sylvatica*** **chromosomes showing the fusion of syntenic blocks of** ***J. effusus*****.** (**a**) Synonymous substitution rate (Ks) determined for *L. sylvatica* genome using CodeML. (**b**) Synteny of *Juncus effusus* to corresponding *L. sylvatica* chromosomes. Note the interruption of synteny near the centromeric regions of most *J. effusus* chromosomes. Genes and CENH3 domains are annotated as blue-purple and red stripes, respectively. Syntenic hits are seen as brown or green (inverted orientation) lines.

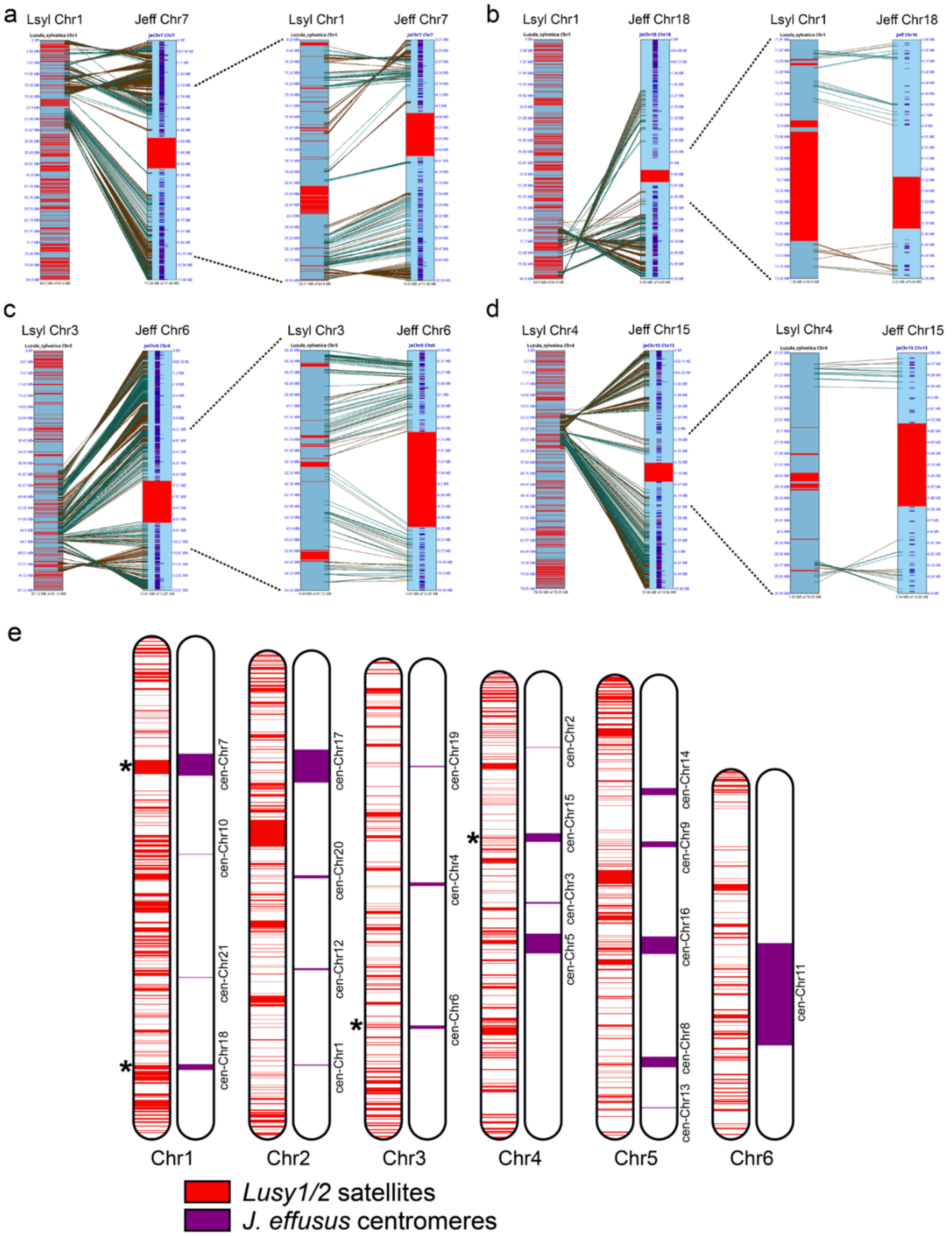

Supplementary Figure 12. Conservation of the ancestral centromere position between *Luzula sylvatica* and *Juncus effusus*. Fine-scale synteny analysis revealed large centromere units that appear conserved between *J. effusus* and *L. sylvatica* genomes. **(a-d)** shows examples of such units, with whole-chromosome view on the left and zoomed-in view on the right. Genes and CENH3 domains are annotated as blue-purple and red stripes, respectively. **(e)** Projections of the coordinates of syntenic blocks closely bordering both sides of centromeres in *J. effu*sus plotted on *L. sylvatica* genome. The space between the two projected centromere-adjacent syntenic blocks is marked in purple to indicate the estimated regions containing ancestral centromeres. The positions of centromeric satellites in contemporary *L. sylvatica* are marked red. Possibly conserved centromeres between *J. effusus* and *L. sylvatica* are highlighted with asterisks.

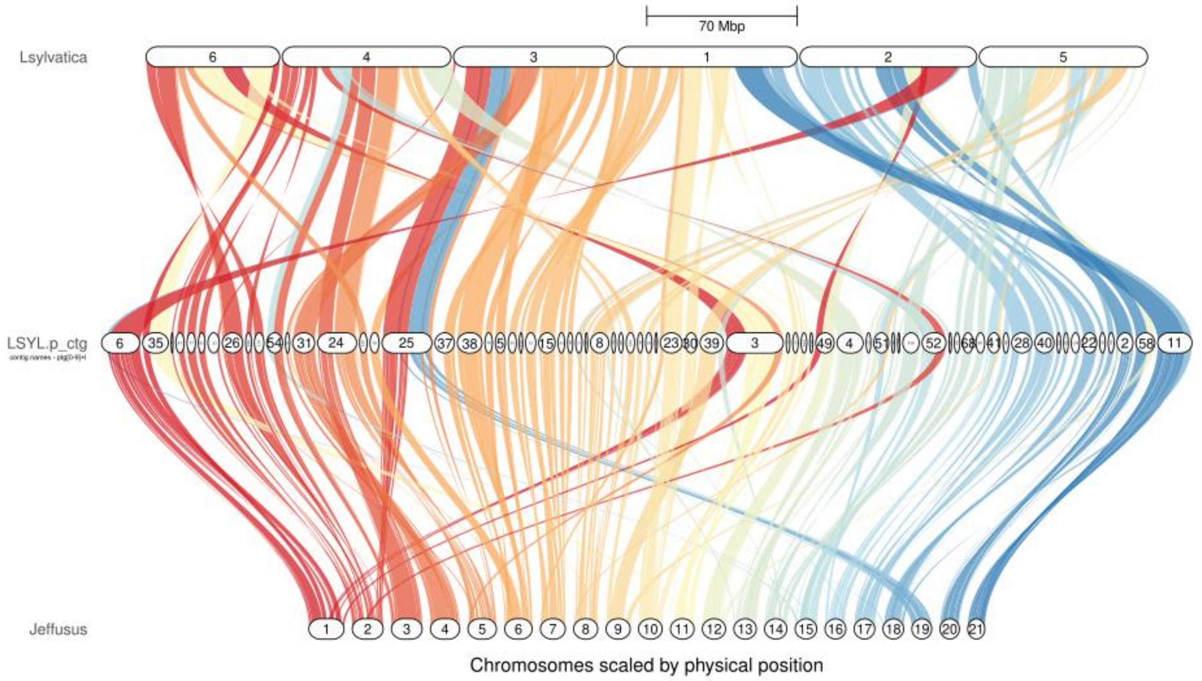

Supplementary Figure 13. Genome synteny between *L. sylvatica* and *J. effusus* contigs. Genome synteny patterns between individual large *L. sylvatica* contigs and *J. effusus* (chromosome-level) contigs shows that the observed fusions and genome rearrangements are not a result of erroneous scaffolding. Source data are provided as a Source Data file.

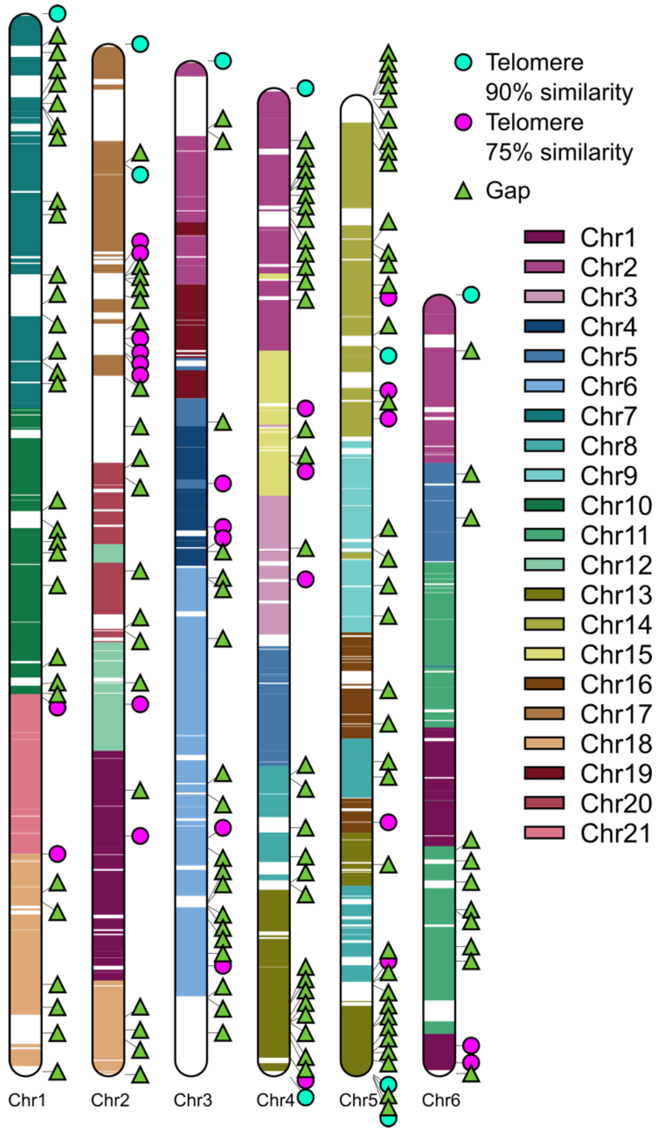

Supplementary Figure 14. Interstitial telomeric sites observed in the genome of *L. sylvatica*. Syntenic regions with the *Juncus effusus* genome are shown as bars on the chromosomes, colors correspond to individual chromosomes. Ends of contigs (assembly gaps) and annotated telomeric regions on the 90% and 75% similarity threshold are marked along the chromosomes. Possible deteriorated ITS were found in the regions bordering (within ~50 kb) both sides of ancestral *J. effusus*-like chromosome 21 (present on *L. sylvatica* chromosome 1) and distal end of ancestral chromosome 8 block on contemporary chromosome 5.

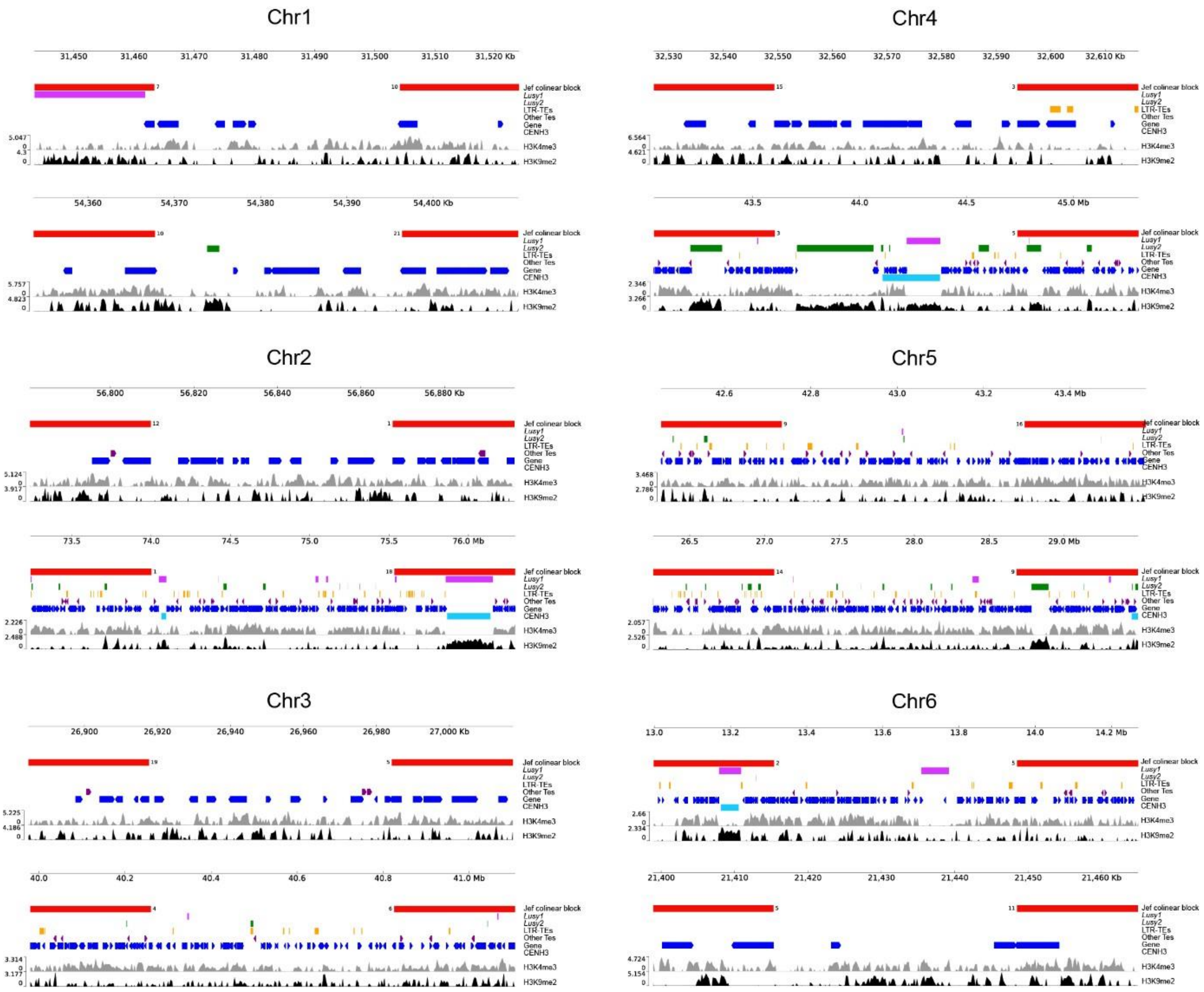

Supplementary Figure 15. Characterization of fusion sites in *L. sylvatica* genome. Similar fusion signatures are shared between some chromosomes, where regions enriched with gene, TE or *Lusy2* repeats are located either up or downstream of the fusion regions.

Supplementary Table 1. Characteristics of the *Luzula sylvatica* genome assembly.

	**Assembly**	**Scaffold**
Total length of contigs (bp)	516,083,138	468,439,054
Largest contig	26,581,686	84,527,883
N50 (bp)	7,692,447	78,954,785
L50	21	4
L90	90	6
Number of unanchored scaffolds		729
GC content (%)		33.01
No. of Gaps		142
N's		2557
AT (%)		66.99

BUSCO	
Complete BUSCOs	1,501 (93.01%)
Complete and single-copy BUSCOs	1,423 (88.2%)
Complete and duplicated BUSCOs	78 (4.8%)
Fragmented BUSCOs	34 (2%)
Missing BUSCOs	79 (5%)
Total BUSCO groups searched	1.614
Average gene length (bp)	1.614
Number of annotated genes	32.644

Supplementary Table 2. Monomer sequence of the satellite DNA of *Luzula sylvatica* genome.

Name	Abundance	Monomer size	Sequence
Lusy1	25.1	124	AAAATCTTGTTTTGAATCGAAAAACAAACTTTCTTAGTGTTTG<br>TGAAATTTGGTGAAAAGTTATGTAGTAAAAGATACGATACAA<br>ATGATCTAAAAACACGTTATTTGAACTCCTCAAAGCGTT
Lusy2.1	3.61	175	TTGTGTGTGTTAGTCTTCAATTACGGAGTATTTTGAGTACGAC<br>ATGAAAATTGAGTATTTTGCTTGTGTTACACTTCAATTTTAACA<br>TTTTTTTAAAAAATATCTTAATTGAATTTTTTGAAGGTGTTAGA<br>CTTCAGTTTTTGCATTTTTAAGAAATTTTTGAAAAAATTGAAAT
Lusy2.2	3.45	174	AACACCTTCAAAAATATCAATTATCATTTTTTCTAAAAAAGAC<br>CAAAATAGTTCTCTAAAATGAGCAAAATACTTCATTTTTCATG<br>TTTTTCTCAAAAAACTCCGTGATTGAAGACTAACACATACAAA<br>TTTCTATTTTTCCTATTTTTTGCAAAAAATCAAAAACTGAAGTC<br>T
LsylSAT3	2.00	182	TATTTTTTGTTAATTCGGGTTCAAAATACATAATTTCCCAAATT<br>CGTTCGCGCTGCTGTATTCCTCTCGTCGAGGCGAGCGATTTTG<br>ATATAAAGATCGTGCCATTTGGAGAAAGATTGAGAAAGTTAC<br>AATGTGACAATCTTCATATTCAATAATTCAAGATGAAATTACC<br>AAAAAAACTG
LsylSAT4	1.05	31	TTTTTTGAAGAAAACCACAAACTAATTTTCT
LsylSAT5	0.06	119	TGATCGTATTCATCGGATAATTAACTATATTTTAGTAAATTCA<br>ATCGTATATTCTGTTAAATTTAATCATATATTTTGCTAAATTCA<br>ATAATATATTTTGCTGAATTATATGAAATTAA
LsylSAT6	0.02	34	TGCAAATCCCGCCAAAAACACAATAGGCGGGATT

Supplementary Table 3. Proportion of repeats in satellite-free CENH3 units of *L. sylvatica* genome.

Name	Count in sat-free CENH3 units	Total length in sat-free CENH3 units	Proportion of sat-free CENH3 units (%)	Proportion in genome (%)
Class_I/LTR/Ty3-gypsy/non-chromovirus/OTA/Athila	392	582.139,00	18	2,6
Class_I/LTR/Ty1-copia/Angela	215	158.332,00	5	10
Class_I/LTR/Ty1-copia/SIRE	58	49.920,00	2	1,6
Class_I/LTR/Ty1-copia/Bianca	48	21.209,00	1	0,7
Class_I/LTR/Ty3-gypsy/chromovirus/Tekay	15	13.567,00	0	0,2
Class_I/LTR/Ty1-copia/Tork	11	10.678,00	0	0,3
Class_I/LTR/Ty3-gypsy/chromovirus/Reina	7	5.811,00	0	0,2
Class_I/LTR/Ty1-copia/Ale	9	4.577,00	0	0,4
Class_I/LTR/Ty1-copia/Ivana	13	2.676,00	0	1
Class_I/LTR/Ty1-copia/TAR	2	46,00	0	0,1
Low_complexity	88	4.628,00	0	0,4
Simple_repeat	643	1.782.330,00	54	3,7
Unknown	2	4,00	0	0

Supplementary Table 4. ***Luzula*** **species, ENA codes and available genome size with their references used for comparative analyses.**

Species	ENA code	Name	1C (Mbp)	Reference
Luzula arcuata (Wahlenb.) Sw.	ERR5554955	Larcu	-	-
Luzula campestris (L.) DC.	ERR5529684	Lcamp	449.88	[1,2]
Luzula elegans Lowe	ERX125774	Leleg	1506.12	[3]
Luzula luzuloides (Lam.) Dandy & Wilmott	ERR5555243	Lluzu	880.20	[4]
Luzula multiflora subsp. f*rigida* (Buchenau) V.I.Krecz.	ERR5555376	Lmtsf	-	-
Luzula nivalis (Laest.) Spreng.	ERR5529777	Lniva	-	-
Luzula nivea (L.) DC.		Lnvea	880.20	-
Luzula parviflora (Ehrh.) Desv.	ERR5554954	Lparv	-	-
Luzula pilosa (L.) Willd.	ERR5554983	Lpilo	224.94	[4,5]
Luzula spicata (L.) DC.	ERR5554743	Lspic	391.20	[5]
Luzula sudetica (Willd.) Schult.	ERR5554826	Lsude	420.54	[1]
Luzula sylvatica (Huds.) Gaudin	ERR5554782	Lsylv	469.44	[5]
Luzula wahlenbergii Rupr.	ERR5554894	Lwahl	-	-

Supplementary references

1. Bačič, T., Dolenc Koce, J. & Frajman, B. Diversification and distribution patterns of *Luzula* sect. *Luzula* (Juncaceae) in the Eastern Alps: a cytogenetic approach combined with extensive herbarium revisions. *Alp Botany* **129**, 149–161 (2019).
2. Bačič, T., Frajman, B. & Dolenc Koce, J. Diversification of *Luzula* sect. *Luzula* (Juncaceae) on the Balkan Peninsula – a cytogenetic approach. *Folia Geobot* **51**, 51–63 (2016).
3. Mukherjee, S., Sen, J. & Sharma, A. K. Cytophotometric DNA estimation in *Luzula* species. *Current Science* **65**, 987–989 (1993).
4. Zonneveld, B. J. M. The DNA weights per nucleus (genome size) of more than 2350 species of the Flora of The Netherlands, of which 1370 are new to science, including the pattern of their DNA peaks. *Forum Geobotanicum* **8**, 24-78 (2019).
5. Šmarda, P. *et al.* Genome sizes and genomic guanine+cytosine (GC) contents of the Czech vascular flora with new estimates for 1700 species. *Preslia* **91**, 117–142 (2019).
