# Supplementary material for: Repeat-based holocentromeres of the woodrush Luzula sylvatica reveal insights into the evolutionary transition to holocentricity
Source: Nat Commun. 2024 Nov 5;15:9565. doi: 10.1038/s41467-024-53944-5 (PMC11538461; doi:10.1038/s41467-024-53944-5)
Supplement: Supplementary file 3 — Description of Additional Supplementary Files [file 41467_2024_53944_MOESM3_ESM.pdf]

Description of Additional Supplementary Files

File Name: Supplementary Data 1
Description: **Individual genome proportion in percentage of the repetitive sequences in Luzula species.** Code names correspond to Larcu: Luzula arcuata, Lcamp: Luzula campestris, Leleg: Luzula elegans, Lluzu: Luzula luzuloides, Lmtsf: Luzula multiflora subsp. frigida, Lniva: Luzula nivalis, Lnvea: Luzula nivea, Lparv: Luzula parviflora, Lpilo: Luzula pilosa, Lspic: Luzula spicata, Lsude: Luzula sudetica, Lsylv: Luzula sylvatica, Lwahl: Luzula wahlenbergii.

File Name: Supplementary Data 2
Description: **Comparative analysis of the shared repetitive sequences between Luzula species genomes**. The different lineages of elements were grouped by cluster in a hierarchical manner following the Repeatexplorer default parameters. Proportions are expressed as percentages. Code names correspond to Larcu: Luzula arcuata, Lcamp: Luzula campestris, Leleg: Luzula elegans, Lluzu: Luzula luzuloides, Lmtsf: Luzula multiflora subsp. frigida, Lniva: Luzula nivalis, Lnvea: Luzula nivea, Lparv: Luzula parviflora, Lpilo: Luzula pilosa, Lspic: Luzula spicata, Lsude: Luzula sudetica, Lsylv: Luzula sylvatica, Lwahl: Luzula wahlenbergii.

File Name: Supplementary Data 3
Description: **Conserved syntenic blocks between L. sylvatica and J. effusus genome.**

File Name: Supplementary Movie 1
Description: Mitotic metaphase in *Luzula sylvatica* showing the attachment of α-tubulin (green) to kinetochore proteins in KNL1 (red) chromosome wide using maximal SIM projections. See individual projections in Suppl. Fig. 7.

File Name: Supplementary Movie 2
Description: Mitotic metaphase in *Luzula nivea* showing the chromosome-wide attachment of α-tubulin (green) on the kinetochore protein KNL1 (red) using maximal SIM projections. See individual projections in Suppl. Fig. 8.

File Name: Supplementary Movie 3
Description: Mitotic metaphase in *Luzula nivea* showing the chromosome-wide attachment of α-tubulin (green) on the kinetochore protein NDC80 (red) using maximal SIM projections. See individual projections in Suppl. Fig. 8.

File Name: Supplementary Movie 4
Description: Mitotic metaphase of *Juncus effusus* showing the restricted attachment of α-tubulin (green) to the kinetochore protein KNL1 (red). See individual projections in Suppl. Fig. 8.

File Name: Supplementary Movie 5
Description: Mitotic metaphase in *Juncus effusus* showing the restricted attachment of α-tubulin (green) to the kinetochore protein NDC80 (red). See individual projections in Suppl. Fig. 8.
