# Supplementary material for: Repeat-based holocentromeres of the woodrush Luzula sylvatica reveal insights into the evolutionary transition to holocentricity
Source: Nat Commun. 2024 Nov 5;15:9565. doi: 10.1038/s41467-024-53944-5 (PMC11538461; doi:10.1038/s41467-024-53944-5)
Supplement: Supplementary file 12 — Reporting Summary [file 41467_2024_53944_MOESM12_ESM.pdf]

nature portfolio

Corresponding author(s): André Marques

Last updated by author(s): Oct 18, 2024

Reporting Summary

Nature Portfolio wishes to improve the reproducibility of the work that we publish. This form provides structure for consistency and transparency in reporting. For further information on Nature Portfolio policies, see our Editorial Policies and the Editorial Policy Checklist.

Statistics

For all statistical analyses, confirm that the following items are present in the figure legend, table legend, main text, or Methods section.

n/a	Confirmed	
☐	☒	The exact sample size (*n*) for each experimental group/condition, given as a discrete number and unit of measurement
☐	☒	A statement on whether measurements were taken from distinct samples or whether the same sample was measured repeatedly
☐	☒	The statistical test(s) used AND whether they are one- or two-sided<br>*Only common tests should be described solely by name; describe more complex techniques in the Methods section.*
☒	☐	A description of all covariates tested
☐	☒	A description of any assumptions or corrections, such as tests of normality and adjustment for multiple comparisons
☐	☒	A full description of the statistical parameters including central tendency (e.g. means) or other basic estimates (e.g. regression coefficient) AND variation (e.g. standard deviation) or associated estimates of uncertainty (e.g. confidence intervals)
☐	☒	For null hypothesis testing, the test statistic (e.g. *F*, *t*, *r*) with confidence intervals, effect sizes, degrees of freedom and *P* value noted<br>*Give P values as exact values whenever suitable.*
☒	☐	For Bayesian analysis, information on the choice of priors and Markov chain Monte Carlo settings
☒	☐	For hierarchical and complex designs, identification of the appropriate level for tests and full reporting of outcomes
☐	☒	Estimates of effect sizes (e.g. Cohen's *d*, Pearson's *r*), indicating how they were calculated

Our web collection on statistics for biologists contains articles on many of the points above.

Software and code

Policy information about availability of computer code

Data collection	HiFi and Hi-C reads obtained through the Darwin Tree of Life database (www.darwintreeoflife.org). Illumina reads from ENA (ENA Browser (ebi.ac.uk)). Images were analyzed using the ZEN software (Carl Zeiss GmbH) and the ZENBlack software (Carl Zeiss GmbH).
Data analysis	Available open source tools used in this study were:<br><br>Augustus (v3.3.3)<br>Bcctools (v0.1.1)<br>Bcftools (v1.9)<br>Bedtools (v2.29.0)<br>Bismark (v0.23.0)<br>bowtie2 (v2.4.4)<br>BUSCO (v5.1.2)<br>CoGe (v7)<br>Cutadapt (v4.7)<br>DANTE_LTR (v0.3.5.2)<br>Deeptools (v3.5.1)<br>Dotter (v0.13.1)<br>EMBOSS (v2024.0419.155605)<br>findGSE_v1.94.R

Geneious (v2023.0.1)
GENESPACE (v1.3.1)
Hifiasm (0.19.8-r603)
Jellyfish (v2.3.1)
Kaks Calculator (v3)
MACS3 (v3.0.0)
minimap2 (v2.26)
ModDotPlot (v0.8.2)
pyGenomeTracks (v3.8)
QUAST (v5.2.0)
RepeatExplorer2 (v2.3.7)
REXdb (v1.0)
SALSA2 (v2.3)
Samtools (v1.9)
SyMAP (v5.0.6)
https://github.com/437364/Repeat-based-holocentromeres-of-Luzula-sylvatica

For manuscripts utilizing custom algorithms or software that are central to the research but not yet described in published literature, software must be made available to editors and reviewers. We strongly encourage code deposition in a community repository (e.g. GitHub). See the Nature Portfolio guidelines for submitting code & software for further information.

Data

Policy information about availability of data

All manuscripts must include a data availability statement. This statement should provide the following information, where applicable:

- Accession codes, unique identifiers, or web links for publicly available datasets
- A description of any restrictions on data availability
- For clinical datasets or third party data, please ensure that the statement adheres to our policy

Data availability
Source data needed to evaluate the conclusions of the paper are provided with this paper and/or as supplementary material. The sequencing data generated in this study have been deposited in the NCBI database under the BioProject ID PRJNA1135980 and are publicly available as of the date of publication. The processed reference genomes, sequencing data, annotations and all tracks data generated in this study are available at DRYAD database: https://doi.org/10.5061/dryad.0gb5mkm8h. The REXdb database Viridiplantae v.3.0 is publicly available at https://github.com/repeatexplorer/rexdb.

Code availability
The original code used in this study is available on GitHub at https://github.com/437364/Repeat-based-holocentromeres-of-Luzula-sylvatica86. Any additional information required to re-analyze the data reported in this paper is available from the corresponding author upon request.

Research involving human participants, their data, or biological material

Policy information about studies with human participants or human data. See also policy information about sex, gender (identity/presentation), and sexual orientation and race, ethnicity and racism.

Reporting on sex and gender	n/a
Reporting on race, ethnicity, or other socially relevant groupings	n/a
Population characteristics	n/a
Recruitment	n/a
Ethics oversight	n/a

Note that full information on the approval of the study protocol must also be provided in the manuscript.

Field-specific reporting

Please select the one below that is the best fit for your research. If you are not sure, read the appropriate sections before making your selection.

☒ Life sciences ☐ Behavioural & social sciences ☐ Ecological, evolutionary & environmental sciences

For a reference copy of the document with all sections, see nature.com/documents/nr-reporting-summary-flat.pdf

Life sciences study design

All studies must disclose on these points even when the disclosure is negative.

Sample size	The size of the sample used was performed according to the requirements for each protocol. For cytological analysis, different root collections

	were collected and analyzed to confirm the reproducibility of the results. For sequencing, sufficient immunoprecipitated DNA was used to sequence 20 million reads.
Data exclusions	No data was excluded form the analysis.
Replication	Replicates were used during the Chipseq analyses for each antibody two replicates were used and because they showed similar patterns, both replicates were mixed for the final analysis. Cytogenetic analyses were performed on several cells, using the best superposition for the final figure.
Randomization	Samples for cytological analysis and chip experiments were randomly selected from two individuals maintained in culture under the same housekeeping conditions.
Blinding	The experiments were performed without knowing the final results.

Reporting for specific materials, systems and methods

We require information from authors about some types of materials, experimental systems and methods used in many studies. Here, indicate whether each material, system or method listed is relevant to your study. If you are not sure if a list item applies to your research, read the appropriate section before selecting a response.

Materials & experimental systems

n/a	Involved in the study	
☐	☒	Antibodies
☒	☐	Eukaryotic cell lines
☒	☐	Palaeontology and archaeology
☒	☐	Animals and other organisms
☒	☐	Clinical data
☒	☐	Dual use research of concern
☐	☒	Plants

Methods

n/a	Involved in the study	
☐	☒	ChIP-seq
☒	☐	Flow cytometry
☒	☐	MRI-based neuroimaging

Antibodies

Antibodies used

Previus designed antibodies:
-rabbit anti-L. elegans CENH3 (LeCENH3; Ma et al., 2016)
-rabbit anti-KNL1 (GenScript, Piscataway, NJ, USA; Oliveira et al. 2024)
-rabbit anti-NDC80 (Biomatik, Cambridge, ON, Canada; Oliveira et al. 2024)

Comercially available antibodies:
-rabbit anti-H3K4me3 (abcam, ab8580)
-mouse anti-H3K9me2 (abcam, ab1220).
-mouse anti αtubulin (Sigma-Aldrich, St. Louis, MO; catalog number T6199)
-goat anti-rabbit Rhodamine Red X (Jackson ImmunoResearch, Suffolk, UK; catalog number: 111–295-144)
-goat anti-mouse Alexa Fluor 488 (ImmunoResearch; catalog number 115-545-166)

Validation

All antibodies were previously validated by:
-Ma et al. (2016) for rabbit anti-LeCENH3
-Oliveira et al. (2024) for rabbit anti-KNL1and rabbit anti-NDC80

or are commercially manufactured and previously validated:
-rabbit anti-H3K4me3 (abcam, Cambridge, UK, catalog number ab8580)
-mouse anti-H3K9me2 (abcam, Cambridge, UK, catalog number ab1220)
- Recombinant rabbit IgG (abcam, Cambridge, UK, catalog number ab172730)
- anti αtubulin by Sigma-Aldrich, St. Louis, MO; catalog number T6199
- anti-mouse Alexa Fluor 488 by Jackson ImmunoResearch; catalog number 115-545-166

Dual use research of concern

Policy information about dual use research of concern

Hazards

Could the accidental, deliberate or reckless misuse of agents or technologies generated in the work, or the application of information presented in the manuscript, pose a threat to:

No	Yes	
☒	☐	Public health
☒	☐	National security
☒	☐	Crops and/or livestock
☒	☐	Ecosystems
☒	☐	Any other significant area

Experiments of concern

Does the work involve any of these experiments of concern:

No	Yes	
☒	☐	Demonstrate how to render a vaccine ineffective
☒	☐	Confer resistance to therapeutically useful antibiotics or antiviral agents
☒	☐	Enhance the virulence of a pathogen or render a nonpathogen virulent
☒	☐	Increase transmissibility of a pathogen
☒	☐	Alter the host range of a pathogen
☒	☐	Enable evasion of diagnostic/detection modalities
☒	☐	Enable the weaponization of a biological agent or toxin
☒	☐	Any other potentially harmful combination of experiments and agents

Plants

Seed stocks	Luzula sylvatica plants were collected in Lake District, UK, and further cultivated under controlled greenhouse conditions (16h daylight, 26 °C, >70% humidity). The ornamental plant Luzula nivea was commercially obtained in Dingers Gartencenter.
Novel plant genotypes	n/a
Authentication	n/a

ChIP-seq

Data deposition

☒ Confirm that both raw and final processed data have been deposited in a public database such as GEO.

☒ Confirm that you have deposited or provided access to graph files (e.g. BED files) for the called peaks.

Data access links<br>*May remain private before publication.*	https://github.com/437364/Repeat-based-holocentromeres-of-Luzula-sylvatica
Files in database submission	all files generated in this study are available under the DRYAD doi: 10.5061/dryad.0gb5mkm8h.
Genome browser session<br>(e.g. UCSC)	no longer applicable.

Methodology

Replicates	Chip experiments had two experimental replicates for each antibody.

Sequencing depth	Each library was sequenced at an approx. 6 genome sequencing depth, 20 million of 1x150bp reads.
Antibodies	Chip experiments were performed for CENH3 (LeCENH3; Ma et al., 2016), H3K4me3 (abcam, ab8580), and H3K9me2 (abcam, ab1220) following Hofstatter et al. (2022) protocol.
Peak calling parameters	macs3 callpeak -t CenH3R1.bam -c inputR1.bam -f BAM -g 800000000 --broad --min-length 1000 --broad-cutoff 0.1 -n CenH3_input_R1 --outdir CenH3_input_R1<br>epic2 -t CenH3R1.bam -c inputR1.bam --chromsizes Lsyl_chr_sizes.tsv -m 0 --output CenH3_input_R1.tsv
Data quality	Quality of peaks were checked by comparison of immunoprecitated DNA enrichment to the controls. Also, high stringency peak filtering approach was chosen to reduce the risk of including false positive CENH3 domains
Software	MACS3, deepTools, bamCompare, Cutadapt , plotProfile, pyGenomeTracks
